# Canadian Conference on Medical Education

**Published:** 2019-04-03

**Authors:** Claire Touchie

**Affiliations:** Chief Medical Education Officer, Medical Council of Canada, Associate Professor, Faculty of Medicine, University of Ottawa

Dear colleagues,

It is with pleasure that I welcome you to the 2019 Canadian Conference on Medical Education (CCME) which is being held in beautiful Niagara Falls, Ontario from April 13 to 16, 2018.

CCME is a proud partnership between 5 national organizations including the Association of Faculties of Canada, the Canadian Association for Medical Association, the College of Family Medicine of Canada, the Medical Council of Canada and the Royal College of Physicians and Surgeons of Canada. The Scientific Program Committee represents all five organizations and carefully prepares a conference program to challenge our views of medical education while providing a collegial environment for scientific exchanges, developing new innovative approaches to learning and teaching, and meeting of like-minded individuals.

The program theme this year is **Disruption Driving Change: Would Sir William Osler Adapt?** With the rapid growth of medical knowledge and the incorporation of technology such as artificial intelligence for diagnosis and management decisions, how will medicine change? How will we adapt our medical programs to keep up with the times? How will all this disruption affect how we provide care to patients? Our plenary themes: Being Human, Being Disruptive and Being Innovative have been chosen to stimulate conversations as we think about the future role of the physician.

Throughout the conference, there will be plenty of opportunities to attend workshops, oral presentations, symposia, posters and various meetings. With its 10^th^ anniversary, White Coat and Warm Art will be a highlight of this year’s conference and again this year, we will host the highly popular Learner Forum. Finally, there will be plenty of networking time for interaction with colleagues as well as a social evening which will highlight the Niagara region’s food and wine.

We look forward to seeing you at the 2019 CCME!

Most sincerely, 
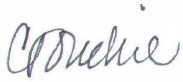

Claire Touchie, MD, MHPE, FRCPC Chief Medical Education Officer, Medical Council of Canada Associate Professor, Faculty of Medicine, University of Ottawa Chair, 2019 Canadian Conference on Medical Education

